# Supplementary material for: Nutritional Quality of Vegetarian and Non-Vegetarian Dishes at School: Are Nutrient Profiling Systems Sufficiently Informative?
Source: Nutrients. 2020 Jul 28;12(8):2256. doi: 10.3390/nu12082256 (PMC7468702; doi:10.3390/nu12082256)
Supplement: Supplementary file 1 [file nutrients-12-02256-s001.pdf]

Supplementary Materials

Binary logistic regression models were used to assess the probability of classification in Nutri-Score class A or SAIN,LIM class 1 according to the category of main dish (Tables S1 and S2), and according to the sub-category of vegetarian dish (Tables S3 and S4), with adjustments for production method (industrial or not) and side dish (“vegetables”, “starches” or “none” for complete dishes).

Table S1. Proportion of dishes in Nutri-Score class A and Nutri-Score classes B, C, D and probability of classification in class A according to the type of side dish, production method and category of main dish

Modality	Class A	Classes B, C, D	Odds-ratio[1]	p-value
Non-vegetarian (ref.)	59,2% (n=396)	40.8% (n=273)		
Vegetarian	66.7% (n=210)	34.3% (n=108)	1.77	0.002
Side dish: starches (ref.)	47.4% (n=186)	52.6% (n=206)		
Side dish: vegetables	78.0% (n=301)	22.0% (n=85)	3.82	<0.001
Side dish: none	57.8% (n=119)	42.2% (n=87)	1.08	0.688
Non-industrial (ref.)	62.5% (n=455)	37.5% (n=273)		
Industrial	59.0% (n=151)	41.0% (n=105)	0.61	0.006

[1]Binary logistic regression model for being classified in class A with three predictors variables: type of side dish, production method and category of main dish.

Table S2. Proportion of dishes in SAIN,LIM class 1 and SAIN,LIM classes 2, 3, 4 and probability of classification in class 1 according to the type of side dish, production method (industrial or not) and category of main dish

Modality	Class 1	Classes 2, 3, 4	Odds-ratio[1]	p-value
Non-vegetarian (ref.)	40.4% (n=270)	59.6% (n=399)		
Vegetarian	65.7% (n=207)	34.3%(n=108)	3.43	<0.001
Side dish: starches (ref.)	9.4% (n=37)	90.6% (n=355)		
Side dish: vegetables	82.9% (n=320)	17.1% (n=66)	50.0	<0.001
Side dish: none	58.3% (n=120)	41.7% (n=86)	8.48	<0.001
Non-industrial (ref.)	47.1% (n=343)	52.9% (n=385)		
Industrial	52.3% (n=134)	47.7%(n=122)	0.95	0.823

[1]Binary logistic regression model for being classified in class 1 with three predictors variables: type of side dish, production method and category of main dish.

Table S3. Proportion of dishes in Nutri-Score class A and Nutri-Score classes B, C, D and probability of classification in class A according to the type of side dish, production method and sub-category of vegetarian dish.

Modality	Class A	Classes B, C, D	Odds-ratio[1]	p-value
VEGAN (ref.)	86.5% (n=115)	13.5% (n=18)		
EGG and/or DP (excl. CHEESE)	64.2% (n=34)	35.8% (n=19)	0.19	<0.001
CHEESE (and/or other DP and/or EGG)	47.3% (n=61)	52.7% (n=68)	0.09	<0.001
Side dish: starches (ref.)	50.7% (n=37)	49.3% (n=36)		
Side dish: vegetables	77.1% (n=84)	22.9% (n=25)	7.05	<0.001
Side dish: none	66.9% (n=89)	33.1% (n=44)	5.27	<0.001
Non-industrial (ref.)	62.5% (n=100)	37.5% (n=60)		
Industrial	71.0%(n=110)	29.0% (n=45)	1.17	0.694

[1]Binary logistic regression model for being classified in class A with three predictors variables: type of side dish, production method and sub-category of vegetarian dish.

Table S4. Proportion of dishes in SAIN,LIM class 1 and SAIN,LIM classes 2, 3, 4 and probability of classification in class 1 according to the type of side dish, production method and sub-category of vegetarian dish.

Modality	Class 1	Classes 2, 3, 4	Odds-ratio[1]	p-value
VEGAN (ref.)	70.7% (n=94)	29.3% (n=39)		
EGG and/or DP (excl. CHEESE)	77.4%(n=41)	22.6% (n=12)	1.04	0.926
CHEESE (and/or other DP and/or EGG)	55.8% (n=72)	44.2% (n=57)	0.29	<0.001
Side dish: starches (ref.)	30.1% (n=22)	69.9% (n=51)		
Side dish: vegetables	89.0% (n=97)	11.0% (n=12)	26.4	<0.001
Side dish: none	66.2% (n=88)	33.8%(n=45)	7.28	<0.001
Non-industrial (ref.)	68.1% (n=109)	31.9% (n=51)		
Industrial	63.2% (n=98)	36.8% (n=57)	1.06	0.899

[1]Binary logistic regression model for being classified in class 1 with three predictors variables: type of side dish, production method and sub-category of vegetarian dish.
